# Comparison of diagnostic and treatment processes among pediatric and adolescents and young adults’ populations suffering from acute lymphoblastic leukemia and lymphomas

**DOI:** 10.3389/fonc.2023.1082789

**Published:** 2023-02-07

**Authors:** Gazala Abdulaziz-Opiela, Anna Sobieraj, Anna Płotka, Barbara Więckowska, Lidia Gil, Katarzyna Derwich

**Affiliations:** ^1^ Poznan University of Medical Sciences, Poznan, Poland; ^2^ Department of Hematology and Bone Marrow Transplantation, Poznan University of Medical Sciences, Poznan, Poland; ^3^ Department of Computer Science and Statistics, Poznan University of Medical Sciences, Poznan, Poland; ^4^ Department of Pediatric Oncology, Hematology and Transplantology, Poznan University of Medical Sciences, Poznan, Poland

**Keywords:** lymphoma, AYA (adolescents and young adults), children, leukemia, healthcare system, adolescents

## Abstract

**Introduction:**

Acute lymphoblastic leukemia (ALL) and lymphomas affect both pediatric and adult populations, therefore, they might be treated by pediatric or adult centers.It has been proven that the prognosis among adolescents and young adults (AYA) is poorer than among children, which remains a subject of research. Many factors are suspected to affect the diagnostic and treatment processes in adolescents and young adults, one of them being the organization of the healthcare system.The aimof the studywas to compare the time intervals between different events on disease trajectory in pediatric and AYA groups suffering from ALL and lymphomas.

**Methods:**

We collected data on 81 patients diagnosed with ALL (50 children and 31 AYAs) and 100 patients diagnosed with lymphomas (50 children and 50 AYAs). Statistical analysis was performed in order to compare the groups.

**Results:**

The results confirmed the hypothesis that the duration of the diagnostic process differs significantly between groups. For patients with ALL, the analyzed time intervals were significantly shorter in the pediatric group than in the AYA group: first contact with a GP - admission to Hematology Department (2 vs. 5 days; pvalue= 0.004), first contact with a GP - treatment (6 vs. 12 days, p-value=0.001), diagnosis - treatment (1 vs. 3 days, p-value=0.003). In the case of patients suffering from lymphomas, the results were similar. The analyzed time intervals were significantly shorter in the pediatric group than in the AYA group: first contact with a GP- diagnosis (21 vs. 40.5 days, p-value<0.0001), first contact with a GP - treatment (27 vs. 65 days, p-value<0.0001). Trend analysis showed that the longer patients had presented symptoms before contacting the primary care physician, the longer they waited for the beginning of treatment both in ALL and lymphomas groups (p-values=0.0129 and 0.0038 respectively).

**Discussion:**

As the diagnostic and treatment processes are longer for AYA patients, actions must be undertaken in order to ensure equality and improve the healthcare system in Poland and possibly other countries.

## Introduction

Acute lymphoblastic leukemia (ALL) and lymphomas affect both pediatric and adult populations. ALL typically occurs among children with incidence rates between 1.8 and 4.5 per 100,000 people per year. The disease accounts for approximately 80% of all leukemia cases in the pediatric population and 56% in the adolescents’ population (1). Among adults, ALL accounts for 0.3% of all new cancer cases with an incidence rate of 1.8 per 100,000 people per year (2).

Lymphomas account for 10% of all childhood cancers and 23% of adolescents’ cancers. In the pediatric population the most common is Hodgkin’s lymphoma. Among non-Hodgkin lymphomas, the most prevalent is Burkitt’s lymphoma. As children age, the incidence of Burkitt’s lymphoma decreases, whereas the incidence of other types increases (3). Hodgkin’s lymphoma is particularly frequent among adolescents, being one of the most common cancers in this group (1, 4). Among adult patients, lymphomas account for 4.6% of all new cancer cases. The most common are non-Hodgkin lymphomas with an incidence rate of 19.0 per 100,000 people per year (2).

Even though the age difference between pediatric and adolescents and young adults (AYA) patients may be small, they are treated by different treatment centers with the usage of different protocols which results in poorer prognosis (5–7). The different treatment outcomes of the same disease in these age groups remains a subject of research. Many components affect the diagnostic and treatment processes in adolescents and young adults, organization of the healthcare system being one of them. It is crucial to assess whether the children treated by specialized pediatric facilities receive better care than AYAs treated by adult departments and, if so, what can be done to improve the situation for AYAs.

## Aims

The aim of the study is to compare the diagnostic and treatment processes in the pediatric (aged between 5 and 18) and the AYA (aged between 18 and 29) groups suffering from ALL and lymphomas. The study investigates the time intervals between the following events: the date of reporting the first symptoms to the primary care physician, the admission to the Hematology Department, the date of receiving a final diagnosis and the date of therapy initiation. The results of the study are supposed to answer whether there are differences between the pediatric and AYA groups and determine where the diagnostic and treatment processes are faster.

## Materials and methods

We gathered data on 81 patients with the diagnosis of acute lymphoblastic leukemia (50 children aged between 5 and 18 and 31 AYAs aged between 18 and 29) and 100 patients with the diagnosis of lymphoma (50 children and 50 AYAs). We received permission from the local Ethics Committee at Poznan University of Medical Sciences to perform this retrospective analysis.

The pediatric group was admitted to the Department of Pediatric Oncology, Hematology and Transplantology at Karol Jonscher’s University Hospital at Poznan University of Medical Sciences in the period 2013-2022. The AYA group was admitted to the Department of Hematology at Heliodor Święcicki’s University Hospital at Poznan University of Medical Sciences in the period 2006-2022.

Due to the lower number of adult patients suffering from acute lymphoblastic leukemia, we were not able to collect enough cases in the time interval 2013-2022. Therefore, we decided to include patients hospitalized earlier. We changed the admission date to 2006 or later among AYA patients. Among the AYA group of 81 patients, 66 were the patients admitted in 2013 or later (81.5%), patients admitted between 2010-2013 were 13.5% and before 2010 - only 5%.

The data on each patient was collected retrospectively from hospitals’ databases and did not require personal contact. The database is anonymous and includes information about the type of symptoms, the duration of symptoms, the date of first contact with a general practitioner, the date of admission to the Hematology Department, the date of diagnosis and the date of beginning of the treatment. Subsequently, statistical analysis was performed using the Microsoft Excel and PQStat v1.6.8 programmes. We calculated the time intervals between the dates and compared them between pediatric and AYA groups.

We performed Pearson’s chi square test and Fisher exact test to analyze the differences between the groups. In order to compare the calculated time intervals, the Mann-Whitney test was performed since the data did not follow normal distribution. The Jonckheere-Terpstra test or Chi-square test for trends were performed to analyze the trends. The significance level was set at less than 0.05.

## Results

### Acute lymphoblastic leukemia

The median age in the pediatric group was 9.4 years and in the AYA group - 22.6 years. Among children, 52.0% were boys and 48.0% were girls. Among adults, 54.8% were men and 45.2% were women. In both pediatric and AYA groups most patients suffered from B-cell ALL. The most common symptoms were general weakness, fever, and lymphadenopathy.

Among pediatric patients with ALL, 78% contacted a general practitioner (GP) when the symptoms lasted less than two weeks. On the contrary, this happened in only 42% of cases in the AYA group. Moreover, 19.3% of AYA patients waited more than 12 weeks before contacting the GP. This never happened in the pediatric group. Adolescents and young adults waited significantly longer before contacting primary care physicians (**Table 1**).

**Table 1:** Demographic table of patients suffering from ALL.

	Pediatric group n=50	AYAs group n=31	
Age [years]			
Range	5.0; 16.0	18.1; 29.4	
Median (Q1, Q3)	9.4 (7.7, 11.6)	22.6 (20.0, 25.4)	
Gender			
Female [%]	48.0	45.2	
Male [%]	52.0	54.8	
ALL type			
B-cell ALL [%]	80.0	87.1	
T-cell ALL [%]	20.0	12.9	
Symptoms			p-value
General weakness [%]	56.0	48.4	0.5046*
Fever [%]	46.0	25.8	0.0691*
Lymphadenopathy [%]	18.0	32	0.1410*
Abnormal bleedings [%]	16.0	13	0.7600**
Weight loss [%]	8.0	6.5	1.0000**
Night sweats [%]	0.0	6.5	0.1436**
Duration of symptoms before contacting a GP			
<2 weeks [%]	78.0	42.0	
2-4 weeks [%]	16.0	29.0	
4-8 weeks [%]	4.0	6.5	
8-12 weeks [%]	2.0	3.2	
>12 weeks [%]	0.0	19.4	
	Trend analysis		0.0002^

Name of the test used: *Pearson’s chi square test **Fisher exact test ^Chi-square statistic for trend.

All analyzed median time intervals were significantly shorter in the pediatric group than in the AYA group (first contact with a GP - admission to Hematology Department, 2 *vs*. 5 days; p-value=0.004; first contact with a GP - treatment, 6 *vs*. 12 days, p-value=0.001; diagnosis - treatment, 1 *vs*. 3 days, p-value=0.003; **Table 2**). As the diagnostic process could improve with time, out of the group of 31 adolescents and young adults diagnosed between 2006 and 2022, we created a smaller group of 18 AYAs diagnosed between 2013 and 2022 and compared them with 50 children diagnosed in the same years. The statistical tests performed on these groups also showed that AYA patients waited significantly longer for the admission to the Hematology department and treatment. The trend analysis showed that the longer the patients had presented symptoms, the longer they waited for the admission to the Hematology Department, the diagnosis, and the beginning of treatment (**Table 2**).

**Table 2:** Comparison of time intervals between pediatric and adolescents and young adults groups suffering from ALL.

Time intervals	Pediatric group Mean ± sd Median(Q1, Q3)	AYA’s group Mean ± sd Median(Q1, Q3)	p-value#	AYA’s subgroup* Mean ± sd Median(Q1, Q3)	p-value#	Trend analysis p-value ##
Between first contact and admission to Hematology Department [days]	5.16 ± 7.87 2 (1, 7)	13.10 ± 17.71 5 (3, 12.5)	0.0040	16.44 ± 21.19 5.5 (2.25, 26.25)	0.0131	0.0249
Between first contact and beginning of treatment [days]	8.42 ± 9.76 6 (3, 11.75)	18.35 ± 17.71 12 (7, 24)	0.0010	21.44 ± 21.12 11 (4.5, 37.5)	0.0152	0.0129
Between diagnosis and the beginning of treatment [days]	1.1 ± 1.42 1 (0, 1.75)	5.45 ± 7.85 3 (1, 6.5)	0.0003	4.56 ± 7.56 1.5 (0.25, 1.5)	0.0392	0.0333

*AYA subgroup of patients diagnosed between 2013 and 2022 (n=18).

Name of the test used: #Mann-Whitney test.

##Jonckheere–Terpstra trend test (trend based on duration of symptoms).

### Lymphomas

The median age of patients was 13.7 years among children and 22.9 years among AYA. Among children, 62% were boys and 38% were girls. Among adults, 52% were men and 48% women. In both age groups, the most common was Hodgkin’s lymphoma. Among non-Hodgkin’s lymphomas, the most common were Burkitt lymphoma in the pediatric group and diffuse large B-cell lymphoma (DLBCL) in the AYA group. The most common symptom in both groups was lymphadenopathy. Half of pediatric patients with lymphomas contacted the primary care physician when symptoms lasted less than two weeks in comparison to 16% of young adults. Moreover, 46% of AYA patients waited more than 12 weeks before contacting the physician. Adolescents and young adults waited significantly longer than children (p-value<0.0001) before contacting a general practitioner (**Table 3**).

**Table 3:** Demographic table of patients suffering from lymphomas.

	Pediatric group n=50	AYAs group n=50	
Age [years]			
Range	5.3; 17.4	18.0; 29.4	
Median (Q1, Q3)	13.7 (9.7, 15.7)	22.9 (19.9, 24.6)	
Gender			
Female [%]	38.0	48.0	
Male [%]	62.0	52.0	
Lymphoma’s types			
Burkitt’s lymphoma [%]	18.0	4.0	
Hodgkin’s lymphoma [%]	58.0	74.0	
Diffuse large B-cell lymphoma [%]	6.0	20.0	
Anaplastic large cell lymphoma [%]	8.0	0.0	
T-cell lymphoblastic lymphoma (T-LBL) [%]	6.0	0.0	
B-cell lymphoblastic lymphoma (B-LBL) [%]	4.0	0.0	
Primary mediastinal large B-cell lymphoma (PMBCL) [%]	0.0	2.0	
Symptoms			
Lymphadenopathy [%]	76	64	0.1904*
Fever [%]	22	20	0.8060*
Night sweats [%]	16	34	0.0377*
Weight loss [%]	16	24	0.3173*
General weakness [%]	14	10	0.5383*
Duration of symptoms before contacting a GP			
<2 weeks [%]	50.0	16.0	
2-4 weeks [%]	16.0	10.0	
4-8 weeks [%]	16.0	16.0	
8-12 weeks [%]	4.0	12.0	
>12 weeks [%]	14.0	46.0	
	Trend analysis		0.00001^

Name of the test used: *Pearson’s chi square test ^Chi-square statistic for trend.

The analyzed median time intervals (first contact with a GP - diagnosis and first contact with a GP - treatment) were both significantly longer among AYA patients than among children (21 *vs*. 40.5 days, p-value <0.0001; 27 *vs*. 65 days, p-value <0.0001). Trend analysis showed that the longer patients had presented symptoms before contacting the primary care physician, the longer they waited for the diagnosis and beginning of treatment (**Table 4**).

**Table 4:** Comparison of time intervals between pediatric and adolescents and young adults groups suffering from lymphomas.

Time intervals	Pediatric group Mean ± sd Median (Q1, Q3)	AYA’s group Mean ± sd Median (Q1, Q3)	p-value#	Trend analysis p-value ##
Between first contact and the diagnosis [days]	27.30 ± 19.41 21 (15.5, 31)	50.7 ± 35.85 40.5 (24.25, 68.75)	<0.0001	0.0309
Between first contact and the beginning of treatment [days]	36.20 ± 24.04 27 (22.5, 47)	70.8 ± 42.27 65 (40.5, 88.25)	<0.0001	0.0038

Name of the test used: #Mann-Whitney test.

##Jonckheere–Terpstra trend test (trend based on duration of symptoms).

## Discussion

In the past, the progress in survivability achieved among AYA patients was inferior to the progress among children (8). Many factors possibly causing the poorer prognosis in that group were discussed, such as differences in dose intensity, protocol schedules and demographic differences due to which adolescents are treated in adult centers instead of pediatric ones (9). Although the trends in survival rates have improved (10), there are still some inequalities between the pediatric and AYA patients. One of them is the difference in enrollments to clinical cancer trials which was highlighted in several research studies (11–13). It has been proven that, in comparison to children, less AYA patients are treated in clinical trials which can result in poorer chances of recovery.

Many researchers claim that the treatment outcomes for adolescents and young adults with ALL are superior when they are treated using pediatric protocols (9), especially at the pediatric departments instead of the adult ones (14). However, the adoption of pediatric regimens continues to be a subject of many discussions (14–16). The majority of patients are treated following adult’s regimens (17). In case of non-Hodgkin’s lymphomas (NHL), treatment’s outcomes are better in pediatric and adolescent patients than in adult ones (18). Usage of pediatric strategies for the AYA population suffering from NHL does not always guarantee a favorable outcome (19). Cooperation of pediatric and adult hematologists and formation of multidisciplinary teams is essential to choose the most adequate treatment strategy (20). Patients from smaller cities must be referred to reference centers specialized in treating ALLs and lymphomas so that they can receive the best standard of care.

The organization of the healthcare system seems to have a significant impact on the inferior outcomes among AYA patients. In the case of the Polish healthcare system the strict age limit of 18 years decides whether an AYA patient is classified as a pediatric or an adult patient. Therefore, even though the age difference between two patients may be small, the one treated by an adult department may face many difficulties due to the overload of the adult healthcare system (21).

The pediatric healthcare system has several advantages over the healthcare available for adults such as better accessibility to a primary care physician or periodic examinations. Moreover, the waiting time for hospitalization is shorter in pediatric departments as the number of patients who require to be admitted is much smaller than at the adult departments. The poorer accessibility to a general practitioner and long waiting times for a specialist consultation may result in poorer outcomes in this group.

It must be acknowledged that the calculated time intervals between different points on the disease trajectory cannot answer the question whether the delay in contacting a general practitioner or admission to the Hematology Department was caused by healthcare system factors as it could be associated with patient decisions as well. Moreover, due to the retrospective character of the study we lacked patients’ feedback and personal feelings about the quality of received healthcare. The time intervals that we calculated are objective parameters, however, they do not deliver full information about the course of the diagnostics and treatment enrollment. Furthermore, the groups of patients suffering from lymphomas presented different distributions of lymphoma’s types. In the pediatric group, 28% of patients were diagnosed with rapid progression lymphomas (Burkitt’s lymphoma, T-LBL and B-LBL). Therefore, the rapid course of the disease itself could have led to a faster diagnostic process and the beginning of the treatment. On the contrary, the majority of AYA patients were diagnosed with Hodgkin’s lymphoma (74%) with a more indolent course.

In order to improve the situation among adolescents and young adults it may be beneficial to form transitional departments for AYAs where both pediatricians and adult doctors could cooperate. This would also reduce the number of patients in adult departments and help older patients suffering from other diseases. All possible actions must be undertaken in order to improve the diagnostic and treatment processes and even the chances of recovery for young people.

## Conclusions

According to the results of the study presented above, adolescents and young adults wait significantly longer to receive medical help. Unfortunately, it causes delays in therapy initiation and may result in poorer prognosis. As the adult healthcare system is overloaded with patients suffering from various diseases, diagnostic and treatment processes are significantly slower. The question arises: what can be done in order to expedite these processes in the AYA group? Formation of transitional departments that take care of adolescents and young adults’ patients might be one of the possible solutions. More research is needed in order to assess the impact of these delays on the treatment outcome. The problem must be further evaluated in Poland and other countries as all patients should receive the best possible care regardless of their age.

## Data availability statement

The raw data supporting the conclusions of this article will be made available by the authors, without undue reservation.

## Ethics statement

The studies involving human participants were reviewed and approved by Bioethics Committee at Poznan University of Medical Sciences, Poznan, Poland. Written informed consent from the participants’ legal guardian/next of kin was not required to participate in this study in accordance with the national legislation and the institutional requirements.

## Author contributions

GA-O, AS and KD designed the study. GA-O and AS collected the data and drafted the manuscript. AP participated in the collection of the data on adult patients. LG coordinated the data collection. BW conducted statistical analysis. KD edited and revised the manuscript. All authors contributed to the article.
